# Illness perceptions among adults aged 18–44 years with ankylosing spondylitis: A qualitative study informed by the common-sense model of self-regulation

**DOI:** 10.1371/journal.pone.0361045

**Published:** 2026-11-10

**Authors:** Di Zhu, Binbin Wang, Yingjin Li, Xiaohan Nie, Haining Yin, Saisai Huang, Qiong Wang, Renju Xu

**Affiliations:** 1 Department of Rheumatology and Immunology, Nanjing Drum Tower Hospital, Affiliated Hospital of Medical School, Nanjing University, Nanjing, China; 2 School of Nursing, Nanjing University of Chinese Medicine, Nanjing, China; 3 Zhongda Hospital, Southeast University, Nanjing, China; The University of Texas Health Science Center at Houston / McGovern Medical School, UNITED STATES OF AMERICA

## Abstract

**Background:**

Ankylosing spondylitis mainly affects people in early and middle adulthood. This period involves important transitions in education, career, relationships, and family. Illness perceptions play an important role in treatment adherence and disease management. Few qualitative studies have examined how Chinese adults perceive and cope with the disease using a clear theoretical framework.

**Objective:**

This study aimed to explore illness perceptions among adults aged 18–44 years with ankylosing spondylitis, informed by the Common-Sense Model of Self-Regulation.

**Methods:**

This was a qualitative descriptive study conducted in a tertiary hospital in Nanjing, China. Sixteen adults aged 18–44 years with ankylosing spondylitis were recruited through maximum variation purposive sampling. Data were collected through face-to-face semi-structured interviews and analyzed using thematic analysis.

**Results:**

Three themes were identified: fragmentation and biases in illness perceptions; disrupted daily life and recurring emotional burden; and negotiating control between professional support and experiential self-management.

**Conclusions:**

Illness perceptions among adults aged 18–44 years with ankylosing spondylitis were dynamic and context-dependent, influenced by symptom experiences, causal interpretations, perceived consequences, and ongoing negotiation between professional guidance and experiential self-management. Illness coherence did not necessarily increase with longer disease duration, and no consistent age-related pattern emerged. These findings offer insight into how adults with AS construct and adapt their illness perceptions, which may guide future patient-centered support.

## Introduction

Ankylosing spondylitis (AS) is a long-term disease that causes serious disability. Its global prevalence ranges from 0.1% to 1.4%, with higher rates seen in North America and Asia [1]. In China, the prevalence is 0.29% [2]. AS mainly affects adults younger than 45, and men are about three times more likely to develop it than women [1,3]. Progressive inflammation and structural damage may lead to spinal ankylosis, functional limitation, and disability over time [4]. Ongoing inflammation also increases the risk of heart disease and raises overall mortality in people with AS compared to the general population [5]. As the disease progresses, some patients experience structural damage and reduced function, which further burdens the disease and makes long-term management more challenging [6].

Current treatments for AS include nonsteroidal anti-inflammatory drugs (NSAIDs), tumor necrosis factor (TNF) inhibitors, interleukin-17 (IL-17) inhibitors, and Janus kinase (JAK) inhibitors [7,8]. Exercise and health education are increasingly recognized as important components of long-term care, but AS remains incurable. Patients need continuous symptom monitoring, treatment decisions, and lifestyle changes throughout the course of the disease. Effective management, therefore, requires not only pharmacological treatment but also adequate patient understanding, shared decision-making, and self-management skills. The insidious onset of the disease, inconsistent information, medication side effects, and uncertainty regarding prognosis may contribute to patient confusion and reduce both treatment adherence and self-management effectiveness [9].

Illness perception refers to the cognitive and emotional responses individuals have toward their disease. It plays an important role in shaping health behaviors [10]. According to the Common-Sense Model of Self-Regulation (CSM), individuals facing health threats develop subjective representations of illness across several dimensions: identity, cause, timeline, consequences, personal and treatment control, emotional reaction, and illness coherence [11]. These perceptions influence coping strategies. The CSM is frequently employed to explain the thoughts and behaviors of patients with chronic diseases.

Descriptive qualitative studies using the CSM indicate that patients who do not understand their disease, misinterpret symptoms, or hold maladaptive beliefs about controllability often experience negative emotions, reduced coping, and challenges with long-term management [12,13]. The CSM was selected for this study because it reflects patients’ multidimensional understanding of disease and clarifies how these perceptions affect emotions and behavior. This approach may help clarify the cognitive processes involved in illness management and provide a theoretical basis for understanding patients’ support needs.

Although previous qualitative studies and syntheses have explored the experiences and perspectives of people with AS [6,14], existing evidence has focused mainly on lived experiences and care needs. Less is known about how adults in early and middle adulthood construct illness representations across CSM dimensions and how these representations are revised over the illness course in response to changing symptoms, treatment experiences, and illness-related information. In particular, the relationships among illness coherence, treatment control, and personal control, as well as possible variation according to age and disease duration, remain insufficiently understood in people with AS. This is relevant for adults aged 18–44 years because AS commonly develops during a life stage characterized by important transitions in education, employment, interpersonal relationships, and family roles [15,16]. Accordingly, this study was informed by the CSM and aimed to explore illness perceptions among Chinese adults aged 18–44 years with AS, with attention to the relationships among illness representations and patterns of variation associated with age and disease duration.

## Materials and methods

### Study design

This study employed a qualitative descriptive design and a data-driven thematic analysis informed by the Common-Sense Model of Self-Regulation (CSM). The CSM was used as a theoretical reference to guide the development of the interview guide and the subsequent interpretation of the findings. It was not applied as a predefined deductive coding framework; rather, themes were first developed from participants’ accounts and were subsequently interpreted in relation to relevant CSM dimensions. Reporting followed the Consolidated Criteria for Reporting Qualitative Research (COREQ) [17].

### Participants

The maximum variation purposive sampling method was employed to achieve diversity and obtain comprehensive information. Variation was considered in gender, age, education, marital status, employment, and disease duration. Participants were recruited from 01/02/2024 to 31/07/2024. Participants were eligible if they were aged 18–44 years and had been diagnosed with AS by a rheumatologist according to the modified New York criteria [18]. The age range of 18 to 44 years was selected because AS typically manifests during this period [19], which is also characterized by significant transitions in education, employment, interpersonal relationships, and family dynamics [15]. Exclusion criteria included the presence of additional psychiatric or cognitive disorders, significant physical limitations that precluded interviews, severe comorbidities such as organ failure or cancer, and hearing or language impairments.

In qualitative research, sample adequacy was assessed through data saturation. Data collection and preliminary analysis proceeded concurrently. Saturation was defined as the point at which successive interviews no longer generated new themes or subthemes or added substantively new information to the developing thematic structure. After the 14th interview, the research team reviewed the emerging codes and themes and judged that relative saturation had been reached. Two additional participants were subsequently interviewed to assess whether further data would modify or expand the existing thematic structure. As these interviews did not generate additional themes or substantively alter the existing themes, data collection was concluded with 16 participants. The sample comprised 9 males and 7 females aged 18–44. Disease duration ranged from 1 to 21 years. Demographic characteristics are summarized in Table 1. Before the beginning of this research, ethical approval was obtained from the Medical Ethics Committee of Nanjing Drum Tower Hospital (Approval No. 202126502). All participants provided written informed consent before enrollment. The study was conducted in accordance with the Declaration of Helsinki.

**Table 1:** Demographic and clinical characteristics of the study participants.

ID	Gender	Age group (years)	Disease Duration (years)	Treatment modality
P1	Female	35–44	6–10	Conventional pharmacological therapy
P2	Female	18–24	≤5	Conventional pharmacological therapy
P3	Female	35–44	≤5	Conventional pharmacological therapy
P4	Male	25–34	>10	Physical therapy
P5	Female	25–34	≤5	No current treatment
P6	Female	18–24	≤5	Biologic therapy
P7	Male	25–34	≤5	Biologic therapy
P8	Male	35–44	>10	Biologic therapy
P9	Female	25–34	≤5	Biologic therapy
P10	Male	25–34	≤5	Biologic therapy; conventional pharmacological therapy
P11	Female	25–34	6–10	Biologic therapy
P12	Male	18–24	≤5	Biologic therapy; traditional Chinese medicine therapy
P13	Male	35–44	>10	Biologic therapy; irregular treatment
P14	Male	25–34	6–10	Biologic therapy
P15	Male	25–34	6–10	Biologic therapy; conventional pharmacological therapy
P16	Male	25–34	>10	Biologic therapy; irregular treatment

### Instrument

Based on a review of relevant literature and the CSM as a theoretical reference, the research team developed an interview guide that covers seven main topics: identity, cause, timeline, consequences, personal/treatment control, emotional representation, and coherence [11]. A group of expert rheumatology clinicians and nursing specialists reviewed the draft interview guide to ensure that its content was valid, clinically relevant, and easy to understand. Pilot interviews were then conducted with two adults who met the inclusion criteria to evaluate the feasibility and practical applicability of the interview guide. Only minor wording adjustments were made after the pilot interviews, and no substantial changes were made to the content or structure of the guide. Therefore, these two interviews were included in the final analysis. The final semi-structured interview guide is provided as S1 File.

### Data collection

All interviews were conducted by the first author, a female nurse with a master’s degree who has received systematic training in semi-structured interview methods and possesses extensive experience in qualitative research. Interviews took place in quiet rooms within the rheumatology outpatient clinic and inpatient ward. All interviews were scheduled after the participants had completed their treatment to avoid disrupting their medical care. During the interviews, access to the rooms was strictly restricted to protect the participants’ privacy. Before each interview, the first author explained the research objectives and procedures to the participants and obtained their written informed consent. Face-to-face interviews were conducted according to a semi-structured interview outline, with each session lasting 30–60 minutes. To guarantee the accuracy of the research data, all interviews were audio-recorded in full using an iFLYTEK smart notebook (model XF-CY-J10E). During each interview, the first author also documented participants’ non-verbal behaviors and emotional expressions in on-site notes, such as crying, smiling, anger, and anxiety. This approach provides a comprehensive understanding of each participant’s experience. During the interviews, the first author adopted a non-directive interviewing approach and refrained from giving participants any leading prompts. Interview skills such as active listening, clarification, and in-depth probing were used to encourage participants to elaborate on their experiences and perceptions. In this way, participants could share their thoughts freely and completely.

### Data analysis

To ensure anonymity, the first author removed all identifying information and assigned unique codes to each participant (P1–P16). After each interview, the first author repeatedly reviewed and transcribed the original audio recordings verbatim in Chinese within 24 hours. To ensure accuracy, the second author cross-checked the transcripts against the original recordings. If segments were unclear or ambiguous, the two authors replayed and discussed them many times until they reached consensus.

Data were analyzed using thematic analysis [20] with MAXQDA 2020. The analysis was primarily data-driven, while the CSM served as a theoretical reference rather than a predefined coding framework. First, the researchers repeatedly read the transcripts to become familiar with the data and identified meaning units relevant to participants’ experiences and perceptions of AS. The researchers generated initial codes inductively from the interview data. Related codes were then compared and clustered to develop potential themes. The researchers repeatedly reviewed the emerging themes against the coded extracts and the full dataset, and merged, separated, refined, or discarded them as needed, following principles of internal coherence and distinction between themes. Once the researchers established a preliminary thematic structure, they examined how the themes corresponded to relevant CSM dimensions, including illness identity, cause, timeline, consequences, personal control, treatment control, emotional representations, and illness coherence. Thus, the CSM was used to interpret and organize the meaning of the findings after theme development rather than to determine the initial coding.

To address variation within the sample, a supplementary cross-case comparison was also conducted after the thematic structure was developed. Age and disease duration were treated as participant characteristics rather than as predefined coding categories. Participants’ accounts were compared across the existing themes to examine whether recurring similarities or differences in illness perceptions were evident according to chronological age or accumulated disease experience. Given the qualitative design and sample size, these comparisons identified patterns of variation across cases rather than establishing formal subgroup effects. All quotations in the manuscript were translated from Chinese to English and verified by bilingual researchers to ensure both semantic accuracy and contextual fidelity.

### Methodological rigor

The trustworthiness of this study’s results was supported by following established qualitative standards, including credibility, dependability, confirmability, and transferability. All interview materials were transcribed verbatim in Chinese by the first author and independently cross-verified by the second author; all ambiguous passages in the statements were resolved through repeated playback and discussion. To promote dependability, data analysis was conducted using thematic analysis with the assistance of MAXQDA 2020, and coding records and decision diaries were maintained. To ensure coding uniformity, the first and second authors independently conducted analyses and compared their results; all differences were discussed with the entire research group until agreement was reached. Additionally, regular team meetings were held to examine how the researchers’ clinical backgrounds could influence data interpretation. During these discussions, the researchers reflected on assumptions arising from their prior clinical knowledge and considered alternative interpretations of the data. The researchers repeatedly checked emerging themes against the original interview transcripts to ensure the interpretations remained grounded in participants’ accounts. To support transferability, this paper provides detailed descriptions of the research environment, participant characteristics (P1–P16), and representative quotations, while protecting participants’ confidentiality through strict de-identification procedures. The completed COREQ checklist is provided in the S2 File.

## Results

A total of 16 participants were included in the analysis. Three main themes and eight subthemes emerged from the interview data. Supplementary cross-case comparisons examined variation by age and disease duration, and relevant patterns are reported within the corresponding themes below. A summary of the thematic structure and its correspondence with the CSM dimensions is presented in Table 2.

**Table 2:** Main themes, related CSM dimensions, subthemes, and illustrative quotations.

Main themes	Related CSM dimension	Subthemes and illustrative descriptions	Illustrative quotations
Fragmentation and biases in illness perceptions	Identity	Figurative symptom perception: participants identified and described AS mainly through bodily sensations, often using vivid metaphors to express pain, stiffness, and immobility.	“It’s hard to describe the pain. It feels like my whole body has been shocked by high-voltage electricity.” (P14, male)
	Cause	External attribution and uncertainty: participants tended to explain disease onset through external causes such as cold exposure, overexertion, fate, or heredity, often with uncertainty.	“Maybe I caught a chill? The first time I felt pain, I was sitting in front of the air conditioner in a very cold room.” (P7, male)
	Timeline	Divergent expectations of disease course: perceptions of disease timeline varied widely, ranging from lifelong incurability to spontaneous recovery or expectations of a rapid cure.	“This disease has no cure. I'll never get better in my whole life!” (P6, female)
	Coherence	Fragmented and dynamically reconstructed illness coherence: participants’ understanding of AS was gradually reconstructed through illness experience and information acquisition, but uncertainty regarding disease progression and long-term management remained in some cases.	“How long do I need to keep taking the medication? Do I have to take it indefinitely, or can I stop once my condition returns to normal?” (P15, male)
Disrupted daily life and recurring emotional burden	Consequences	Flare-dependent disruption of functioning: the disease burden became particularly salient during flare-ups or after structural damage, disrupting mobility, self-care, work, and family roles.	“Most of the time, I feel just like everyone else. But when my illness gets worse, my legs become very stiff, and it is hard for me to take the bus, ride the subway, or climb stairs. I cannot go to work during these times.” (P14, male)
	Emotional representation	Fluctuating emotional experiences: emotional responses shifted over time and included fear, frustration, anxiety, loneliness, and inferiority.	“I had never even heard of this disease. I felt like my entire life had collapsed!” (P2, female)
Negotiating control between professional support and experiential self-management	Treatment control	Conditional trust in professional treatment: participants’ confidence in professional treatment was strengthened by perceived treatment effectiveness and clear, consistent clinical guidance, but could be weakened by insufficient communication or conflicting recommendations.	“I don't know whether I should take the medication or not. Different doctors give different treatment approaches... I received two opposite answers.” (P15, male)
	Personal control	Autonomous regulation based on bodily experience: participants used bodily feedback and accumulated experience to adjust exercise, daily activities, and, in some cases, medication use.	“Going to the hospital just results in the same types of medicine being prescribed. It’s no different if I buy them myself. When the pain is severe, I take a few extra painkillers and then stop when I feel better.” (P16, male)

### Theme 1: Fragmentation and biases in illness perceptions

Participants described fragmented and sometimes inconsistent illness representations across symptom perception, causal attribution, timeline expectations, and illness coherence. These representations varied across participants and were reflected in different ways of identifying symptoms, explaining disease onset, anticipating the course of AS, and integrating illness-related experiences and information.

### Figurative symptom perception

Participants’ understanding of the condition often began with bodily sensations. Labeling symptoms differently appeared to be the main way of identifying the disease. While most participants recognized symptoms such as pain, morning stiffness, and impaired function, the subtle and difficult-to-define nature of these symptoms often caused distress. Consequently, they frequently employed metaphors such as ‘electric,’ ‘insects,’ ‘rocks,’ or ‘pillars’ to describe their experiences. One participant described the pain as high-voltage electricity: *“It’s hard to describe the pain. It feels like my whole body has been shocked by high-voltage electricity.” (P14, male).* Others used similarly vivid metaphors to describe their symptoms, including *“It feels like insects are crawling and biting deep within your muscles and bones.” (P9, female), “My lower back feels like a rock. I can't move it.” (P10, male),* and *“It feels like the pillar supporting my back has collapsed. I can't turn over or get out of bed……” (P7, male).*

### External attribution and uncertainty

Because the pathogenesis of AS remains incompletely understood, most participants attempted to identify the cause of their illness by recalling possible triggering factors. One participant stated, *“Maybe I caught a chill? The first time I felt pain, I was sitting in front of the air conditioner in a very cold room.” (P7, male).* Additionally, another participant commented, *“It probably happened because I was tired after dancing.” (P13, male).* Uncertainty was also a prominent feature of participants’ causal interpretations. Some referred to fate or destiny, as reflected in the statement, *“What should you do if God wants you to get sick?” (P1, female).* Others suspected a hereditary basis but remained confused about how heredity might operate: *“I think I got it from my mum because my uncle also has back pain. But my older brother and sister don't have this problem. Why me?” (P14, male).*

### Divergent expectations of disease course

Participants had different views on the timeline of AS. Some viewed it as an incurable, lifelong disease. For example, one participant conveyed a pessimistic outlook: *“This disease has no cure. I'll never get better in my whole life!” (P6, female).* Some believed the disease had resolved when the pain subsided. One participant stated, *“If the pain doesn't come back, I've fully recovered.” (P16, male)*. Two participants anticipated a short-term cure. One remarked, *“I've heard that biologics work well, so I'm hoping for a quick recovery.” (P3, female).* Another participant believed that the condition would improve spontaneously with age and without intervention: *“Around the age of 40, AS will stop progressing.” (P11, female).*

### Fragmented and dynamically reconstructed illness coherence

Participants’ understanding of AS was not static but was continuously adjusted and reconstructed through their symptom experiences, diagnosis, treatment experiences, and acquisition of illness-related information. As their experiences with the illness accumulated, some participants gradually moved from a vague understanding of the illness toward greater recognition of its chronic, recurrent nature and the need for long-term management. One participant stated, *“At first, I didn't know AS could be this serious. I thought I would probably get better after taking some medication, but gradually I realized that it might actually be quite a serious condition.” (P2, female).* Another participant reinterpreted previous bodily experiences after acquiring more illness-related knowledge. *“It was only later that I learned about the concept of morning stiffness. Looking back now, I realize that what I experienced at that time was morning stiffness.” (P10, male).*

Cross-case comparison showed that longer disease duration did not consistently correspond to greater illness coherence. Although some participants with longer illness experience described clearer recognition of the chronic and recurrent nature of AS, uncertainty regarding disease progression and long-term management remained evident in others. One participant remained uncertain about the duration of treatment, asking, *“How long do I need to keep taking the medication? Do I have to take it indefinitely, or can I stop once my condition returns to normal?” (P15, male)*.

### Theme 2: Disrupted daily life and recurring emotional burden

Participants described substantial disruption to daily functioning and recurrent emotional burden associated with AS. Functional consequences were particularly apparent during symptom flares or after structural damage, while emotional responses varied across the illness course and included fear, anxiety, loneliness, and reduced self-worth.

### Flare-dependent disruption of functioning

The early symptoms of AS were often subtle and slowly progressive. Many participants did not fully recognize the burden of the disease until an acute flare resulted in functional impairment. One participant described a marked loss of independence during exacerbations. *“When I'm not having a flare, everything is normal. But once it starts, I can't comb my hair, fasten my bra, tie my shoelaces, or cut my nails. Even something as simple as using the toilet becomes impossible.” (P1, female).* Another participant emphasized the impact on mobility and work participation*. “Most of the time, I feel just like everyone else. But when my illness gets worse, my legs become very stiff, and it is hard for me to take the bus, ride the subway, or climb stairs. I cannot go to work during these times.” (P14, male).* Disruption also extended to family roles. *“I'm perfectly fine when I'm not having a flare-up, but when I am, I can't even pick up my child!” (P8, male).* For some participants, the severity of AS became apparent only after structural damage had occurred. One participant noted, *“My daily life used to be almost completely unaffected, but then the bone in my hip joint fused together. Now, simple tasks like getting dressed, putting on socks, or bending over to pick something up require a lot of effort.” (P11, female).*

Cross-case comparison suggested that the functional consequences of AS did not follow a clear age-related pattern. Instead, age-related variation was more apparent in the life contexts in which functional disruption occurred, including work, family responsibilities, mobility, and basic daily activities.

### Fluctuating emotional experiences

In the early period following diagnosis, participants described negative emotions such as fear, despair, anxiety, and a sense of life disruption. One participant recalled, *“How could I get this disease at such a young age? All I wanted to do at that moment was jump off the hospital building” (P5, female).* Another participant with shorter disease duration stated, *“I had never even heard of this disease. I felt like my entire life had collapsed!” (P2, female).* For some participants, emotional distress diminished when symptoms were controlled but re-emerged during pain flares. As one participant with longer disease duration explained, *“I have the feeling that I am a healthy person most of the time, but when the pain comes, I become very anxious” (P13, male).* Participants with persistent pain or functional impairment also described more enduring psychological burden. One participant reported, *“Since falling ill, I have been staying at home every day. My family doesn't understand my condition, so I constantly feel very lonely. Since the diagnosis, I have felt inferior no matter where I am” (P8, male).*

Cross-case comparison suggested that the nature and timing of emotional responses varied with disease duration. Participants with shorter disease duration tended to emphasize the emotional shock associated with diagnosis and the initial confrontation with a chronic illness, whereas some participants with longer disease duration described distress as recurring in response to pain flares, persistent functional limitations, and disruption to social roles. However, these patterns overlapped across participants and did not represent distinct disease-duration groups.

### Theme 3: Negotiating control between professional support and experiential self-management

Participants described control over AS as involving both professional treatment and experiential self-management. Treatment control reflected confidence in medical treatment and professional guidance. In contrast, personal control reflected participants’ efforts to regulate symptoms, daily activities, and management decisions based on bodily experience.

### Conditional trust in professional treatment

Participants generally regarded professional healthcare as an important resource for controlling the illness. In particular, when symptoms worsened or became difficult to manage on their own, they were more likely to seek medical help and follow professional guidance. One participant stated, *“I really had no choice but to go to the hospital and see a doctor... I took my medication on time every day... I followed everything the doctor told me” (P7, male).* Another participant explicitly expressed trust in physicians’ professional judgment: *“I just need to follow the treatment plan the doctor gives me. If the doctor gives you a plan, it must be based on examinations, so I just follow it” (P6, female).* Similarly, some participants sought professional help promptly when symptoms emerged or changed. One participant stated, *“Whenever I feel any discomfort, I go to the hospital right away” (P14, male).* Such trust could also be reinforced by perceived treatment effectiveness. After receiving biologic therapy, one participant reported, *“After the first injection, I felt completely different... I felt no different from a normal person” (P11, female).*

However, participants’ trust in professional treatment was conditional on the quality and consistency of professional guidance. Clear explanations of treatment plans strengthened confidence in care. One participant explained, *“A good doctor will communicate with you and discuss medication... They need to explain clearly to patients why this medication is being used and what advantages it has over conventional treatment.” (P5, female).* In contrast, inconsistent recommendations could generate uncertainty. Another participant stated, *“I don't know whether I should take the medication or not. Different doctors give different treatment approaches... I received two opposite answers.” (P15, male).*

Cross-case comparison therefore suggested that participants with shorter disease duration tended to place greater emphasis on professional guidance, although this pattern was not uniform. Continued reliance on professional care among participants with longer disease duration indicated that disease duration alone did not determine perceptions of treatment control; perceived treatment effectiveness and the clarity and consistency of professional communication also appeared important.

### Autonomous regulation based on bodily experience

Personal control was reflected in participants’ efforts to regulate symptoms, physical functioning, and daily life according to their own bodily experiences. Participants adjusted exercise, rest, and daily activities in response to pain, stiffness, and other bodily feedback. Such experiential regulation was particularly evident in some participants with longer disease duration. One participant with longer disease duration described developing an activity pattern through repeated observation of his physical condition. *“I worked it out based on my own physical condition... Every time I exercise, my whole body feels much more relaxed.” (P16, male).* Among some participants with longer disease duration, experiential self-management also extended to medication decisions. One participant with longer disease duration described reducing the physician-recommended injection frequency from once a week to once every two weeks after his symptoms improved. *“I'm no longer in pain, all my indicators are coming down, and there is indeed financial pressure as well.” (P13, male).* Another participant also decided whether to take medication according to his symptoms. *“I take medication when it hurts, and I don't take it when it doesn't.” (P16, male).*

Cross-case comparison suggested that some participants with longer disease duration drew more strongly on accumulated bodily experience when making self-management decisions. However, this pattern was not uniform, as longer disease duration did not invariably lead to greater reliance on experiential control. Overall, participants’ sense of personal control was grounded in ongoing bodily observation and experiential judgment, which supported adaptive adjustments to daily activities but could also extend to autonomous treatment decisions.

## Discussion

Informed by the CSM, this study explored illness perceptions among adults aged 18–44 years with AS and identified three themes: fragmentation and biases in illness perceptions; disrupted daily life and recurring emotional burden; and negotiating control between professional support and experiential self-management. The findings suggest that illness perceptions were dynamic and context-dependent, changing with symptom experiences, treatment experiences, and illness-related information. No consistent age-related pattern emerged, while variation by disease duration was more apparent but not uniform. Beyond organizing patient experiences within CSM dimensions, the present study further highlights their dynamic interrelationships: longer disease duration did not necessarily correspond to greater illness coherence, while perceived consequences, emotional burden, and perceptions of control could be repeatedly reshaped over the course of the illness.

This study found that adults aged 18–44 years with AS showed perceptual biases in symptom perception, causal attribution, and expectations of disease course, consistent with findings from several previous studies [21,22]. AS is characterized by an insidious onset, fluctuating symptoms, a prolonged disease course, and complex pathogenesis [23]. In light of the present findings, these disease characteristics may make it more difficult for patients to accurately identify and perceive their illness, leading them to rely more heavily on their own bodily experiences and previous life experiences when making sense of the disease, thereby contributing to perceptual biases. Compared with previous research, the present study further suggests that these perceptual biases do not occur in isolation but may be jointly shaped by bodily experiences, previous life experiences, and illness-related information, with different dimensions of illness perception becoming intertwined. Importantly, participants’ accounts suggest that bodily experiences may serve as an important reference point for interpreting not only symptoms but also disease causes, expected disease course, and controllability. Thus, changes in bodily sensations may contribute to the simultaneous revision of several illness representations rather than influencing a single CSM dimension in isolation. These findings suggest that healthcare professionals could consider dynamically assessing illness perceptions among adults aged 18–44 years with AS, with particular attention to specific biases in symptom perception, causal attribution, and expectations of disease course. CSM-informed interventions commonly include assessment of patients’ existing illness perceptions followed by individualized education and feedback [24]. Visual approaches may also facilitate comprehension of health information and modification of illness beliefs [25]. Accordingly, structured questioning, individualized health education, and visual aids could be considered as potential approaches to addressing inaccurate or fragmented illness perceptions.

Beyond biases in individual illness dimensions, fragmentation was also evident in how participants connected different aspects of AS into a coherent overall illness representation, with illness coherence being continually reconstructed over the course of the disease. Previous research has likewise indicated that illness perceptions are repeatedly reappraised and adjusted in response to new illness-related information and coping outcomes [26]. Davenport et al. [27] found that complex and difficult-to-understand information may hinder the development of coherent illness representations. In contrast, clear and easily processed information may facilitate the establishment of illness coherence. Building on these findings, the present study further suggests that longer disease duration does not necessarily correspond to greater illness coherence; rather, integrating symptom experiences, treatment feedback, and illness-related information acquired at different stages may deserve greater attention. This suggests that illness coherence may depend less on the simple accumulation of illness experience than on whether new experiences and information can be integrated into patients’ existing illness representations. In this sense, longer disease duration may provide more opportunities for reconstruction without necessarily producing a more coherent illness representation. Therefore, during long-term follow-up, healthcare professionals could monitor changes in patients’ illness coherence and provide staged, accessible professional guidance at key points such as diagnosis, symptom changes, and treatment adjustment. Newly acquired illness-related information could be linked with patients’ existing illness perceptions, while patients’ understanding of this information could be checked to support the development of more coherent illness representations.

Perceptions of illness consequences were strongly context-dependent, becoming particularly salient when symptoms interfered with daily activities, work, and family roles. Previous studies have similarly shown that AS-related pain and functional limitations can affect patients’ daily activities, work capacity, and family and social roles [28]. The present study further suggests that perceptions of illness consequences are not constant but vary according to changes in symptom burden and functional status. When symptoms are stable, the perceived impact of the disease on everyday life may recede into the background. In contrast, activity limitations during disease flare-ups may make perceived illness consequences more salient. This pattern suggests that patients may appraise illness consequences largely through their current functional experiences rather than through a stable evaluation of AS’s long-term impact. Moreover, the absence of a consistent age-related pattern suggests that, within the 18–44-year age range, differences in perceived consequences may be more closely related to life-role contexts, such as education, employment, family responsibilities, and independent daily functioning, than to chronological age itself. Therefore, healthcare professionals could explore the specific effects of AS on patients’ daily activities, work, and family roles during follow-up and provide individualized activity and energy-management guidance based on the main areas of limitation. Rehabilitation support may also be considered where appropriate to address activity limitations associated with disease flare-ups.

Emotional responses likewise fluctuated across the illness course rather than following a stable or progressively diminishing pattern. During the early stage of the disease, participants could experience strong negative emotions such as fear and anxiety. These emotional responses tended to ease as symptoms came under control but could re-emerge when pain recurred. Wilson et al. [29] similarly reported that disease symptoms and their continuing impact on daily life are important sources of emotional distress. The present study further suggests that emotional burden in people with AS does not necessarily diminish progressively with longer disease duration but may fluctuate repeatedly with changes in disease status and perceived threat. This indicates that emotional adaptation to AS may not follow a simple linear trajectory as disease duration increases. Rather, recurrent pain or renewed functional disruption may reactivate patients’ appraisal of illness threat, bringing previously attenuated emotional responses back to the foreground. The fluctuation in emotional burden may therefore be closely linked to the context-dependent appraisal of illness consequences observed in this study. Within the CSM framework, emotional responses are closely related to individuals’ ongoing appraisal of illness threat and its consequences and may change as this appraisal is revised over time [26]. Therefore, healthcare professionals could consider monitoring changes in patients’ emotional status over time. Beyond the period around diagnosis, emotional burden may also warrant assessment during disease flare-ups, symptom worsening, and treatment adjustment, with appropriate emotional support or specialist referral considered when necessary.

Patients generally viewed professional treatment as an important source of disease control, although their trust in treatment was conditional. Clear treatment effects and consistent professional guidance could strengthen perceived treatment control, whereas conflicting recommendations could create uncertainty. Previous research has similarly emphasized the importance of accessible, trustworthy treatment information; effective patient–healthcare professional communication; and patient involvement in treatment decisions in axial spondyloarthritis (axSpA) [30]. As treatment options such as biologic therapies continue to expand, patients also need to weigh treatment effectiveness, mode of administration, adverse effects, and personal preferences. In addition, the present study further suggests that trust in professional treatment is dynamic and may be continuously adjusted according to perceived treatment effectiveness and the clarity and consistency of the professional information received. Thus, treatment control appeared to arise not simply from receiving professional treatment, but from patients’ ongoing appraisal of treatment feedback and the credibility and consistency of treatment-related information. Therefore, at key treatment points, particularly when initiating or switching biologic therapy, healthcare professionals could consider providing continuous, consistent, and individualized information on treatment effectiveness, risks, and monitoring, while using shared decision-making and decision aids to support patients in weighing treatment options.

Meanwhile, the Internet, social media, and online patient communities have become important sources of medical information for patients with rheumatic diseases. Information from inconsistent, unreliable, or conflicting sources may affect patients’ judgments about treatment effectiveness and professional recommendations, thereby weakening their perceived treatment control [31]. Patients’ ability to evaluate and integrate online health information may depend on digital health literacy, thereby influencing how such information contributes to the formation and revision of treatment-control perceptions [32]. Healthcare institutions could consider providing professionally reviewed AS-related information through digital channels, such as official WeChat accounts or mini-programs, to offer patients more consistent and verifiable treatment information.

Personal control was reflected in patients’ use of bodily feedback and accumulated experience to guide self-management. Participants adjusted exercise, rest, and daily activities according to their physical condition, with some individuals with longer disease duration relying more strongly on their own judgment. Studies by Ingram et al. [33] and Paul et al. [34] similarly suggest that patients with axSpA gradually develop individualized disease-management strategies through repeated attempts and bodily feedback. The present study further found that personal control based on bodily experience was reflected not only in regulating daily life but also in medication use and other treatment decisions. Taken together with the findings on treatment control, personal control and treatment control did not appear to represent mutually exclusive sources of control. Rather, participants appeared to continually recalibrate the balance between professional guidance and experiential judgment according to treatment feedback, symptom changes, and accumulated bodily experience. Professional treatment could remain an important source of perceived control even among patients with longer disease duration, while accumulated experience could simultaneously strengthen confidence in autonomous regulation. This distinction may help explain why longer disease duration was associated with greater reliance on experiential judgment in some participants but not in others. Therefore, healthcare professionals could consider integrating patients’ bodily experiences with symptoms and objective disease assessment when providing individualized guidance, while using shared decision-making to clarify appropriate boundaries for exercise and treatment adjustments.

## Study limitations

This study has several limitations. First, recruitment from a single tertiary hospital, where most participants were already receiving specialist care, may limit transferability and underrepresent patients with poorer healthcare access or lower treatment engagement. Second, although disease duration and treatment modality were recorded, other relevant clinical characteristics were not systematically collected. Third, age- and disease-duration comparisons were intended to identify patterns across cases rather than formal subgroup effects and should be interpreted cautiously. Fourth, although the analysis was primarily data-driven, use of the CSM in interpretation may have introduced confirmation bias; independent coding, reflexive discussion, and repeated checking against transcripts were used to reduce this risk. Finally, self-reported interview data may be subject to recall bias. In addition, social desirability bias cannot be excluded because all interviews were conducted by the first author, a nurse working at the same hospital where the participants received care. Participants may have been inclined to provide responses that they perceived as more acceptable to a healthcare professional.

## Conclusions

This study suggests that illness perceptions among adults aged 18–44 years with AS are dynamic and context-dependent. Importantly, longer disease duration did not necessarily correspond to greater illness coherence. At the same time, perceptions of consequences, emotional burden, treatment control, and personal control were continually reshaped by changing symptoms, treatment experiences, and accumulated bodily experience. No consistent age-related pattern was identified. These findings extend existing descriptions of AS patient experience by highlighting the dynamic relationships among illness perception dimensions over the course of the illness.

## Supporting information

S1 File Semi-structured interview guide used in this study. (DOCX)

S2 File Completed 32-item COREQ checklist. (DOCX)
