# Supplementary material for: Illness perceptions among adults aged 18–44 years with ankylosing spondylitis: A qualitative study informed by the common-sense model of self-regulation
Source: PLoS One. 2026 Nov 10;21(11):e0361045. doi: 10.1371/journal.pone.0361045 (PMC13750555; doi:10.1371/journal.pone.0361045)
Supplement: Supplementary file 1 [file pone.0361045.s001.docx]

**Supporting Information 1**

**Interview Guide**

**Identity:** "What symptoms or problems have you experienced since being diagnosed with AS?"

**Cause:** "In your opinion, what factors or causes led to the development of your AS?" **Timeline:** "How do you perceive your condition will change or progress over time?" **Consequences:** "What kind of impact has AS had on your life?"

**Personal and treatment control:** "How do you believe AS should be effectively controlled or treated?"

**Emotional Representation:** "What emotional changes or psychological experiences have you had since your diagnosis?"

**Coherence:** "Overall, how do you personally understand or make sense of AS as a disease?"
