# Supplementary material for: Illness perceptions among adults aged 18–44 years with ankylosing spondylitis: A qualitative study informed by the common-sense model of self-regulation
Source: PLoS One. 2026 Nov 10;21(11):e0361045. doi: 10.1371/journal.pone.0361045 (PMC13750555; doi:10.1371/journal.pone.0361045)
Supplement: Supplementary file 2 [file pone.0361045.s002.docx]

Supporting Information 2

Consolidated criteria for reporting qualitative studies (COREQ): 32-item checklist

| No | Item | Guide questions/description |
| --- | --- | --- |
| Domain 1: Research team and reflexivity | | |
| Personal Characteristics | | |
| 1. | Interviewer/facilitator | DZ. |
| 2. | Credentials | DZ (MSc), BW (BSc), YL (MSc), XN (BSc), HY (MSc), SH (PhD), QW (BSc). |
| 3. | Occupation | DZ (Charge Nurse), BW (Nurse Manager), YL (Student), XN (Registered Nurse), HY (Charge Nurse), SH (Attending Physician), QW (Registered Nurse). |
| 4. | Gender | Female. |
| 5. | Experience and training | The team members have all received rigorous study and training in qualitative research, and DZ has some experience with qualitative interviews. |
| Relationship with participants | | |
| 6. | Relationship established | No. The interviewer (DZ) had no prior relationship with the participants before the study commenced. |
| 7. | Participant knowledge of the interviewer | Participants were informed that the interviewer was conducting a study to explore illness perceptions among adults with ankylosing spondylitis. Before each interview, the study objectives and procedures were explained, and written informed consent was obtained. |
| 8. | Interviewer characteristics | The interviewer was a nurse working at the study hospital. The research team held regular discussions to reflect on how their clinical backgrounds and prior knowledge could influence data interpretation. |
| Domain 2: Study design | | |
| Theoretical framework | | |
| 9. | Methodological orientation and Theory | A qualitative descriptive design was used, with data-driven thematic analysis informed by the Common-Sense Model of Self-Regulation (CSM). The CSM guided the development of the interview guide and the subsequent interpretation of findings; it was not used as a predefined coding framework. |
| Participant selection | | |
| 10. | Sampling | Purposive sampling. |
| 11. | Method of approach | Potential participants were approached face-to-face by the first author in outpatient and inpatient clinical settings. The researcher introduced the study's objectives and significance in person and invited eligible patients to participate. |
| 12. | Sample size | 16 |
| 13. | Non-participation | Zero. We used purposive sampling to select interviewees, and after contacting them, all agreed to participate in the interviews. |
| Setting | | |
| 14. | Setting of data collection | Interviews were conducted in quiet, private rooms in outpatient and inpatient clinical settings. Access to the interview rooms was strictly restricted during the sessions to protect participants' privacy and provide a comfortable environment for open communication. |
| 15. | Presence of non-participants | No. |
| 16. | Description of sample | See Table 1. |
| Data collection | | |
| 17. | Interview guide | An interview guide was drafted and piloted; see supporting information 1. |
| 18. | Repeat interviews | None. |
| 19. | Audio/visual recording | All interviews were audio-recorded. |
| 20. | Field notes | Yes. |
| 21. | Duration | 30–60 minutes. |
| 22. | Data saturation | Yes. |
| 23. | Transcripts returned | Yes. |
| Domain 3: Analysis and findings | | |
| Data analysis | | |
| 24. | Number of data coders | 2. |
| 25. | Description of the coding tree | See Table 2. |
| 26. | Derivation of themes | Themes were derived inductively from the interview data through thematic analysis. The CSM was not used as a predefined coding framework. After a preliminary thematic structure had been developed, the themes were interpreted in relation to relevant CSM dimensions. |
| 27. | Software | MAXQDA 2020. |
| 28. | Participant checking | Yes. |
| Reporting | | |
| 29. | Quotations presented | Yes. |
| 30. | Data and findings consistent | There is strong consistency between the raw data and the reported findings. Each of the three main themes and eight sub-themes is supported by several representative and verbatim quotations from the 16 participants. These quotations are integrated into the Results section to provide transparent evidence for the researchers' interpretations of the patients' lived experiences. |
| 31. | Clarity of major themes | Yes. |
| 32. | Clarity of minor themes | Yes. |
